# Evaluation of the effect of transcutaneous electrical nerve stimulation (TENS) on whole salivary flow rate

**DOI:** 10.4317/jced.51828

**Published:** 2015-02-01

**Authors:** Hersheal Aggarwal, Mohit Pal-Singh, Hemant Mathur, Sowmya Astekar, Pranay Gulati, Shruta Lakhani

**Affiliations:** 1 MDS Oral Medicine & Radiology – Private Practice; 2 Professor – Dept. of Oral Medicine & Radiology, Pacific dental college, Udaipur; 3 Asst. Professor- Dept. of Oral Medicine & Radiology, Pacific dental college, Udaipur; 4 Asst. Professor - Dept. of Oral Medicine & Radiology, Institute of dental sciences, Bareilley; 5 BDS - Private practice

## Abstract

Background: Saliva plays a critical role in maintaining oral homeostasis; it modulates the ecosystem through lubrication of the alimentary bolus, protection against microorganisms, buffer and repair of the oral mucosa, and helps in dental re-mineralization. Various local and systemic factors such as medications, radiation therapy, systemic conditions, etc. can lead to reduction in salivary flow. A decrease in salivary function, known as Xerostomia, increases a patient’s risk for caries and other oral infections. Palliative management of Xerostomia includes wetting agents such as ice chips, drugs and saliva substitutes. Systemic agents stimulate salivary flow but often have unfavorable side effects. Newer modalities like transcutaneous electrical nerve stimulation (TENS), which has fewer side effects, have been used to stimulate salivary flow. The aim of the present study was to assess and evaluate the effect of TENS on whole salivary flow rates in healthy adult subjects. Study design: A total of 80 healthy adult subjects were enrolled in the study. Unstimulated and stimulated saliva (using TENS) was collected for 5 minutes and the mean salivary flow rates were calculated. Data obtained was analyzed using the SPSS (Statistical package for social sciences) version 15. Students ‘t’ test was employed for comparative analysis. Results: Sixty-five of the 80 subjects demonstrated an increase in the salivary flow rate on application of TENS. Twelve subjects demonstrated a mild reduction in the salivary flow rates. Seven subjects experienced transient mild twitching of facial musculature as side effects. Conclusion: Significant increase in salivary flow rates was observed on application of TENS with minimal or no side effects.

** Key words:**Stimulated saliva, whole salivary flow, TENS.

## Introduction

Saliva is a critical fluid necessary for oral health. The 3 major pairs of salivary glands along with 300-500 minor salivary glands produce about 1.5 L of whole saliva daily. At rest, secretion ranges from 0.25 to 0.35 ml/min. Sensory, electrical or mechanical stimuli can raise the secretion rate to 1.5 ml/min (1,2).

It has been known for over 150 years that the nerves to salivary glands control the secretion of saliva (3). Salivary secretion is regulated by a three component reflex arch including: (a) afferent receptors and nerves, (b) a central connection and processing nucleus (salivation center), and (c) an efferent reflex arm constituted by parasympathetic and sympathetic nerves bundles (4). The afferent nerves carry impulses from the periphery to the salivation center in the medulla oblongata, which in turn directs signals to the efferent part of the reflex arch leading to salivation (5). Given the autonomic control of salivary secretion, the electrical stimulation of one of the components of the salivary reflex arch can potentially lead to enhancement of salivary secretion (4).

Transcutaneous electrical nerve stimulation (TENS) consists of application of low frequency, pulsed electrical currents. These electrical currents are transmitted via surface electrode pads placed on the skin surface and potentially stimulate the peripheral nerves to produce various physiological effects (6). The first TENS units were developed in the year 1965 after the publication of the gate control theory by Melzack and Wall (7). Since 1965, TENS has become known worldwide and is also considered to be one of the most common therapeutic resources used in clinical practice for the relief of chronic and acute pain. However, in recent times, many researchers have observed that in addition to the analgesic effects of TENS, it may also be used to increase salivary flow by stimulating the peripheral nerves.

The aim of the present study was to assess and evaluate the effect of TENS on whole salivary flow rates in healthy adult subjects.

## Material and Methods

Patients attending the routine out patient department of the institute during the period of March 2012 to April 2012, who were apparently healthy, were included in this prospective cross sectional study. Exclusion criteria included patients with a history of salivary gland pathology, patients wearing active pacemakers, patients suffering from systemic diseases and conditions, patients currently taking medications for any conditions, those with a history of radiation to the head and neck region, patients with a history of psychiatric disorders and pregnant women. All the included subjects were explained the procedure and consent was obtained. A total of 80 subjects, 40 males and 40 females were included in the study with an age range between 20 and 50 years. Ethical clearance for the study from the ethical clearance committee of the institute was obtained.

All the subjects were explained the design of the study and were asked to refrain from eating, drinking, chewing gum, smoking, and oral hygiene procedures for at least 1 hour prior to the appointment.

**Figure 1:**
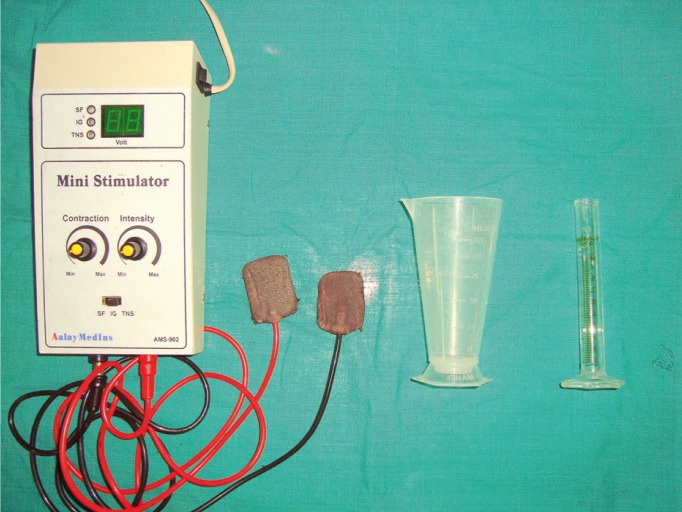
TENS unit used in the study.

**Figure 2:**
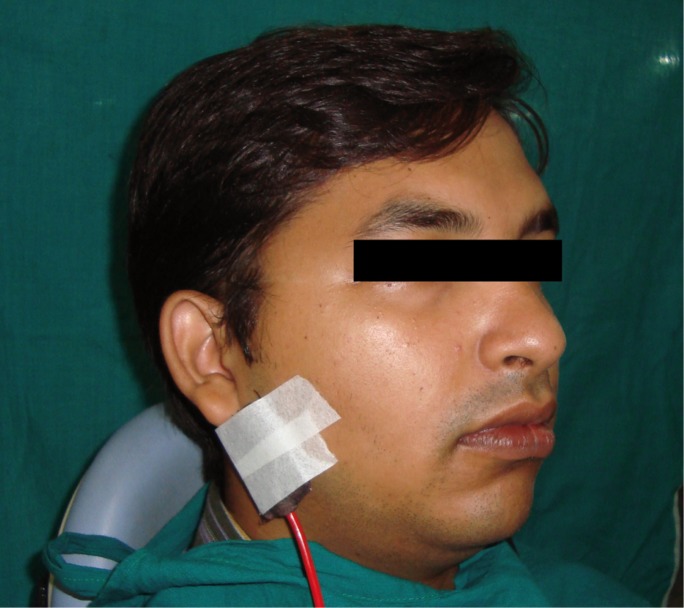
Patient positioned with surface electrode pads.

The TENS unit employed for this study was Anlaya MedIns – AMS-902 (Fig. 1). The surface electrode pads were placed externally on the skin, overlying the parotid glands, with the TENS unit in the ‘off’ position. The subjects were made to sit in an upright position, with the head inclined slightly forward (Fig. 2). They were asked to swallow saliva first and then instructed to stay motionless, so that the saliva would collect passively in the anterior region of the floor of the mouth. With low forced spitting, unstimulated saliva was then collected for five minutes, in a graduated test tube fitted with a funnel. The unit was preset at a frequency of 100 Hz and a pulse width of 100-150µs. After a gap of about two minutes, the TENS unit was activated and the amplitude was gradually increased to a maximum tolerable level of patient (6). Stimulated saliva was collected for five minutes in a separate graduated test tube and the flow rate was compared with the unstimulated salivary flow rate.

Subjects served as their own controls. A log of adverse events was kept. Student‘t’ test was used for group comparisons. Correlation Analysis was performed to assess the relationship between measurements. For all the tests, *p* value of 0.05 or less was considered statistically significant.

## Results

The mean unstimulated salivary flow rate was 1.25 ml/min., the mean stimulated salivary flow rate was 1.41 ml/min ( Table 1). Sixty five of the 80 subjects showed an increase in the salivary flow rate upon application of TENS. Twelve subjects showed no increase in the salivation, while three subjects showed a decrease in the salivary flow. These findings showed an approximately 13% (0.16 ml/min) increase in the mean salivary flow rate on application of TENS. Statistical evaluation showed this increase to be highly significant (*P*<0.001) ( Table 1).

**Table 1:**
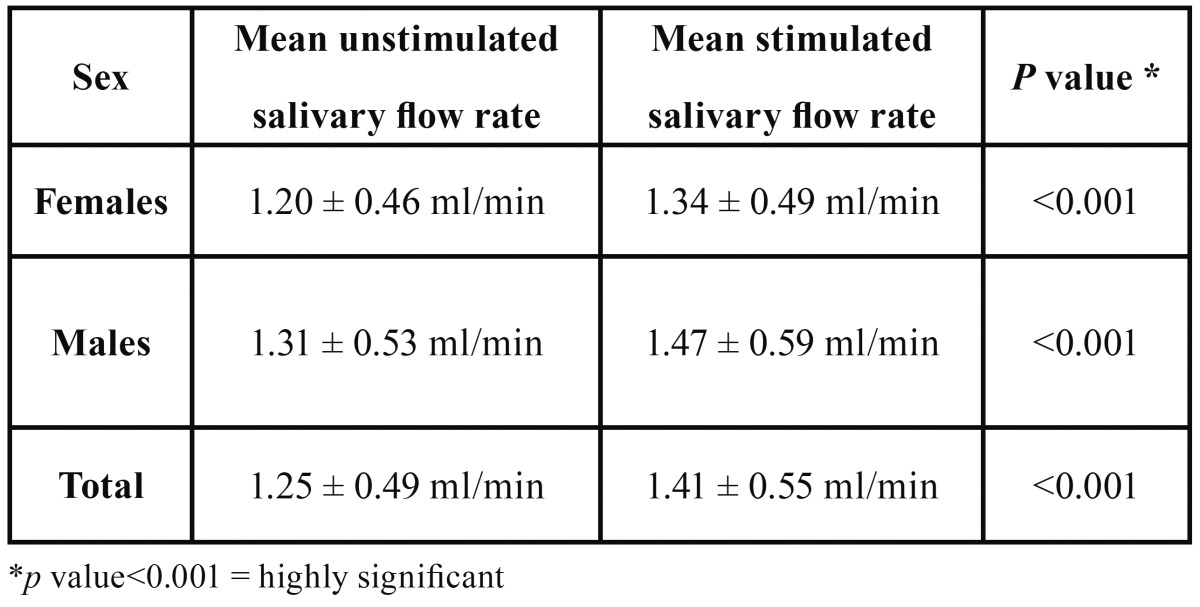
Mean stimulated and Unstimulated salivary flow rates.

Also, the increase in salivary flow rates for males was slightly higher but not statistically significant as compared to females.

Subjects across all age groups showed statistically significant increase in stimulated salivary flow rates ( Table 2).

**Table 2:**
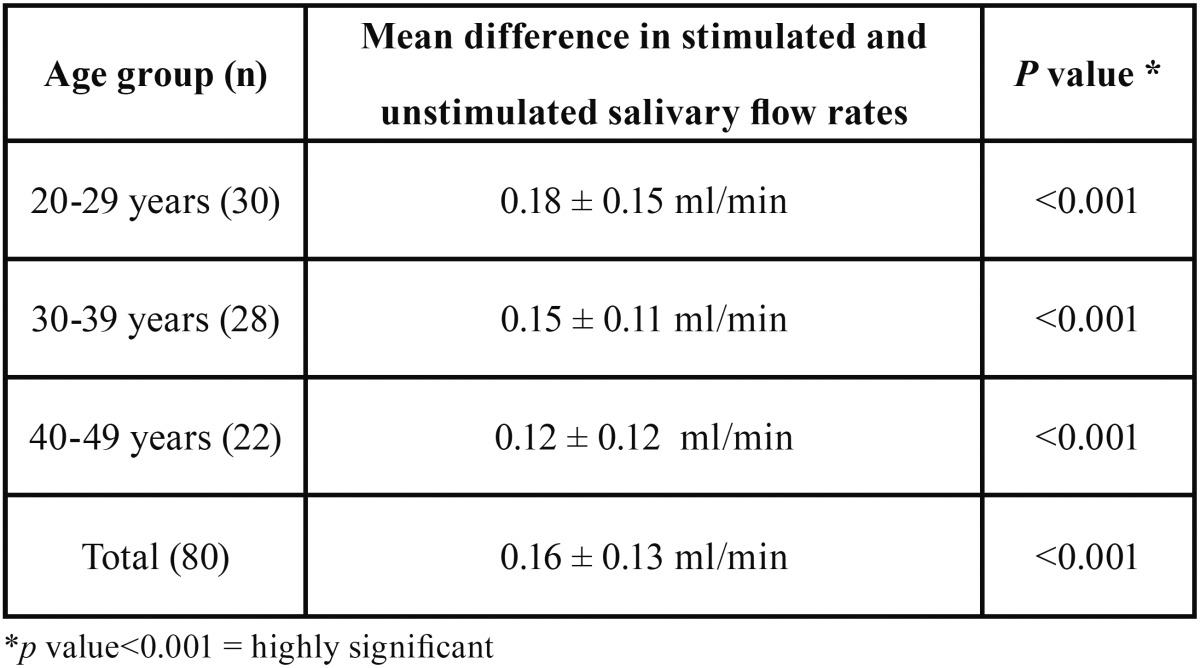
Comparison of increase in salivary flow rates.

Seven subjects experienced mild twitching of the facial musculature during the application of TENS, which ceased once TENS was deactivated. The subjects reported no other adverse effects.

## Discussion

The application of electric current through the oral mucosa to the afferent neuronal pathway causes neuroelectrical stimulation of the salivary glands and this has been reported to increase the production of saliva and to reduce the symptoms of xerostomia (8). The effect of transcutaneous electrical nerve stimulation (TENS) has been evaluated in stimulating salivary flow and it was found to be effective even in patients with xerostomia secondary to radiation therapy for head and neck cancer.

In the present study, 65 out of 80 subjects responded with an increase in the salivary flow rate following the application of TENS. There was approximately a 13% increase in the mean salivary flow rate. A study conducted by Hargitai *et al.* (9) also demonstrated similar results where in 15 out of the 22 subjects had demonstrated increased salivary flow rate. In another study conducted by Pattipati *et al.* (10) in 2013, showed an increase in salivary flow rate on application of TENS, and more so, this increase in salivary flow was pertinent even one hour after the application of TENS in a select group of individuals.

In the present study, 12 of the 80 subjects showed no variation in the salivary flow on application of TENS. It has been shown in literature that 21% – 22% of the population demonstrates no parotid flow even when measured over five minutes (11). Since the electrode pads were placed over the parotid gland, it can be conceived that in these patients, there was no enhancement in parotid flow and the volume of whole saliva collected was from the other components such as submandibular gland, sublingual gland, and gingival crevicular fluid (GCF).

In three patients, the salivary flow was decreased with the application of TENS. This finding was also similar to a study conducted by Hargitai *et al.* (9). The cause for this may involve the frequency and intensity settings. The stimulus perceived by the brain may be painful and the salivary reflex is enhanced when nociceptive input reaches the brain via trigeminal sensory nuclei. Not all preganglionic parasympathetic fibers are necessarily facilitated; some may be inhibited thus leading to the decrease in salivary flow rate (9).

The effectiveness of TENS in stimulating salivary flow was not dependent on age. The literature has shown that salivary flow does not diminish with age, and our results are in agreement with this observation (11).

The mechanism by which the TENS unit worked on the parotid gland may be that it directly stimulates the salivary secretion arc. Salivary secretion is regulated by a neuronal mechanism consisting of a reflex arch. This neuronal mechanism has three basic components; (1) afferent receptors and nerves which carry impulses generated by masticatory and gustatory actions; (2) a central connecting and processing center (salivatory nucleus); and (3) an efferent neuronal pathway consisting of parasympathetic and sympathetic nerve bundles that separately but in a coordinated manner innervate the blood vessels and acini of their target glands leading to regulation of salivary secretion (12). It is believed that afferent nerves carry impulses from the periphery to the salivary nuclei (salivation center) in the medulla oblongata, which in turn directs signals to the efferent part of the reflex arch leading to initiation of salivation (12).

Electrostimulation of neuromuscular structures has proven to be of therapeutic potential in several areas of medical sciences such as pacemakers, phrenic stimulators, etc. and since the autonomic nervous control of salivary secretion has been proven, similar electrostimulation of the neuronal component of salivary secretion can potentially be of therapeutic use for enhancing salivary secretion in salivary hypofunction. Hence, application of electric impulses to one or more of the three components of the salivary arch should in theory improve salivary secretion and, thereby, lessen the long-term effects of hyposalivation (13).

Studies that demonstrate the use of extra-oral transcutaneous electric nerve stimulation (TENS) over the parotid gland was reported to increase saliva production in healthy individuals and patients with radiation-induced xerostomia, suggest that TENS might directly stimulate the auriculotemporal nerve (efferent pathway) that supplies the secretomotor drive to the parotid gland (8,9).

The greatest advantage of this technique over other sialogogues is the almost complete absence of any lasting local or systemic side effects. The main drawback of this technique is the extraoral device, as placement of the device may not always be suitable. Future researches should assess as to how long the increase in salivary flow lasts after turning off the TENS unit, the increase in salivary flow rate in patients suffering from dry mouth, patient acceptability, and comparison with other sialogogues.

This study demonstrated that a TENS unit was effective in increasing parotid gland salivary flow in most healthy adult subjects. Side effects were minimal and transient. Further studies in a cohort of xerostomia sufferers are warranted. If such studies reflect findings similar to the present study, TENS may be a useful treatment option in the management of salivary gland hypofunction when other therapies have failed or are contraindicated.
